# Effect of Transition Elements on the Thermal Stability of Glassy Alloys 82Al–16Fe–2TM (TM: Ti, Ni, Cu) Prepared by Mechanical Alloying

**DOI:** 10.3390/ma14143978

**Published:** 2021-07-16

**Authors:** Nguyen Thi Hoang Oanh, Do Nam Binh, Dung Dang Duc, Quyen Hoang Thi Ngoc, Nguyen Hoang Viet

**Affiliations:** 1 School of Materials Science and Engineering, Hanoi University of Science and Technology, No 1 Dai Co Viet, Hai Ba Trung, Hanoi 100000, Vietnam; oanh.nguyenthihoang@hust.edu.vn (N.T.H.O.); binhdn@moit.gov.vn (D.N.B.); quyen.hoangthingoc@hust.edu.vn (Q.H.T.N.); 2 School of Engineering Physics, Hanoi University of Science and Technology, No 1 Dai Co Viet, Hai Ba Trung, Hanoi 100000, Vietnam; dung.dangduc@hust.edu.vn

**Keywords:** Al-based amorphous alloy, thermal stability, mechanical alloying, solid-state transformations

## Abstract

In the present study, the thermal stability and crystallization behavior of mechanical alloyed metallic glassy Al_82_Fe_16_Ti_2_, Al_82_Fe_16_Ni_2_, and Al_82_Fe_16_Cu_2_ were investigated. The microstructure of the milled powders was characterized by scanning electron microscopy (SEM), X-ray diffraction (XRD), and differential scanning calorimetry (DSC). The results showed remarkable distinction in thermal stability of the alloys by varying only two atomic percentages of transition elements. Among them, Al_82_Fe_16_Ti_2_ alloy shows the highest thermal stability compared to the others. In the crystallization process, exothermal peaks corresponding to precipitation of fcc-Al and intermetallic phases from amorphous matrix were observed.

## 1. Introduction

It is widely known that metallic glasses are novel engineering alloys, which exhibited properties that were different from conventional crystalline materials. The unique properties of metallic glass were originally derived from the random atomic arrangement of metallic glasses [1,2,3,4]. Owing to the absence of grain boundaries and crystal defects typically found in crystalline materials, amorphous alloys exhibit excellent mechanical properties. The tensile strength of melt-spun Al–ETM–LTM amorphous ribbons exceeded 1200 MPa [5] (early transition metal (ETM), late transition metal (LTM)), which was approximately three times that of conventional aluminum alloys [2]. The Al-based metallic glasses have a great potential application in the automotive industry, aerospace and military fields, requiring high corrosion resistance, high strength, and a high-specificity elastic modulus [6]. Among the aluminum alloys, iron aluminides containing more than 80% Al are promising candidates for structural applications due to high specific strength and excellent corrosion resistance at elevated temperatures under oxidizing, carburizing, and sulfurizing atmospheres [7]. The addition of Fe in an Al matrix by mechanical alloying technique results in a super-saturated solution formation during the milling process and in amorphous Al–Fe alloys in final production [6]. Normally, metallic glasses are metastable materials at room temperature. Under a suitable heating process, the atoms rearrange to form crystalline or quasicrystalline phases [1,4,8,9,10]. The crystallization behavior of Al–Ni–La ternary [11] and Al–Fe–Ni–La quaternary [12] systems indicated a three-stage process with primary crystallization of the fcc–Al. It has been noticed that the homogeneous dispersion of nanocrystalline fcc–Al particles in the residual amorphous matrix increase in tensile strength up to 1560 MPa, as reported in [13]. Despite the remarkable improvement of tensile strength of the amorphous materials, the limited ductility can be incorporated into the amorphous matrix through the dispersion of nanometric phases [4,9,14]. N. Bassim et al. [15] studied the crystallization behavior and microstructure development upon annealing amorphous melt-spun ribbons Al_84_Y_9_Ni_5_Co_2_. The crystallization onset temperature of this alloy, at a heating rate of 20 K/min, was 292 °C. J. Q. Wang et al. [16] investigated the crystallization behavior of as-quenched Al_88_Ni_9_Ce_2_Fe_1_ melt-spun ribbons. The first crystallization reaction with precipitation of nanocrystals had an onset temperature of 155.2 °C. Additionally, Viet et al. [17] studied the crystallization kinetics of Al–Fe and Al–Fe–Y amorphous alloys synthesized by mechanical alloying. It was reported that the crystallization onset temperatures can be increased with an increase in the Fe content and substitution of Y for Al. However, only a few works have studied the thermal stability and crystallization behavior of the Al–Fe–TM (transition metal (TM)) alloys prepared by mechanical alloying (MA) to date. Some practical techniques synthesize amorphous alloys with metastable equilibrium microstructures, such as mechanical alloying (MA), melt spinning, gas atomization, and rapid quenching techniques [4,9,18,19,20,21,22,23,24]. Among them, MA is a versatile method owing to its simplicity and ease of synthesis. The MA products are produced in powder form, which is very helpful for the sintering process. In addition, MA also extends solid solubility in many binary systems, which are normally immiscible in the solid state or even in the liquid state [9,25,26,27].

In this work, the thermal stability of metallic glasses alloys with a nominal composition of Al_82_Fe_16_Ti_2_, Al_82_Fe_16_Ni_2_, and Al_82_Fe_16_Cu_2_ prepared by mechanical alloying is investigated. The effect of minor alloying additions (Ti, Ni, Cu) on crystallization and thermal stability of Al–Fe alloys is also examined.

## 2. Materials and Methods

82Al–16Fe–2TM (TM: Ti, Ni, Cu) amorphous alloy powders were prepared by mechanical alloying from elemental blend powders in a Fritsch Pulverisette-6 (Fritsch, Idar-Oberstein, Germany) planetary ball mill at a rotation rate of 300 rpm, according to our previous work [28]. Hardened steel balls were used at a 20:1 ball to powder weight ratio. Stainless steel vials were used to contain the powder and milling balls, and 50 mL of n-hexane was added as a control agent to prevent sticking phenomena. Before milling, the vials were sealed and evacuated. The milling processes were interrupted every 30 min to prevent excessive heating. In order to investigate the thermal stability, three alloy powders, after milling for 10, 40, 50, and 60 h in amorphous state, corresponding to Al_82_Fe_16_Ti_2_, Al_82_Fe_16_Ni_2_, and Al_82_Fe_16_Cu_2_, respectively, were chosen.

The morphology of milled powders was characterized by field-emission scanning electron microscopy (FE-SEM) using a JEOL JSM-7600F (JEOL Ltd., Tokyo, Japan). Phase analysis was done by X-ray diffraction (XRD) in a SIEMENS D5000 diffractometer (Siemens, Berlin, Germany) using Cu Kα radiation (λ = 1.5405 Å). The XRD parameters were: 2θ range of 20 to 80°; a step size of 0.03°; scanning speed 1° per min. Particle size distribution of amorphous powders was tested by the Laser Scattering Particle Size Distribution Analyzer LA-960 (Horiba Ltd., Kyoto, Japan). The MDI Jade version 6.5 (associated with the ICDD PDF2 database, 2007, Newtown Square, PA, USA) was used for the peaks matching the reference sample. The refined lattice parameters of crystallization phases were evaluated via Profex (version 4.3.2a, released 30 March 2021, Solothurn, Switzerland), a graphical user interface for Rietveld refinement of powder X-ray diffraction data with the program BGMN [29]. The thermal stability of as-milled powders was studied by differential scanning calorimetry (DSC) in a Netzsch STA 449C–QMS 403C Thermal Analyzer System (Netzsch Gerätebau GmbH, Selb, Germany). The non-isothermal DSC studies were carried out at a heating rate of 20 K/min under a continuous flow of purified argon gas flow. Specimens after heating in the calorimeter were investigated by XRD.

## 3. Results

**Figure 1:**
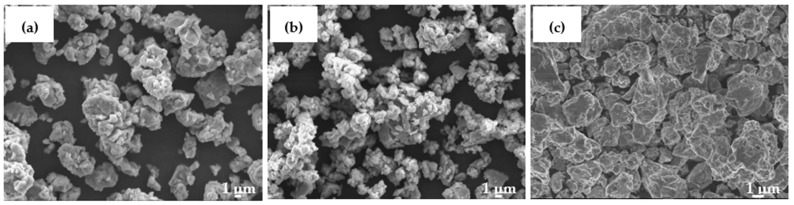
FE-SEM micrographs of (**a**) Al_82_Fe_16_Ti_2_, (**b**) Al_82_Fe_16_Ni_2_, and (**c**) Al_82_Fe_16_Cu_2_ powders after 40, 50, and 60 h of milling, respectively.

**Figure 2:**
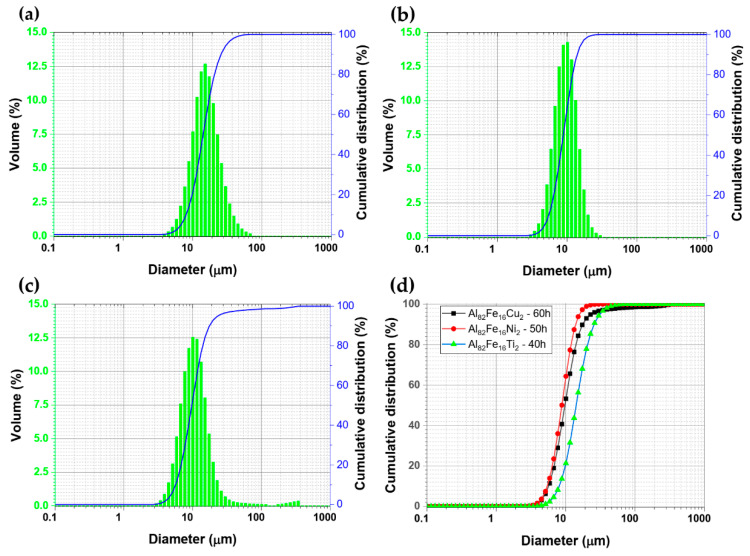
Particle size distribution of (**a**) Al_82_Fe_16_Ti_2_, (**b**) Al_82_Fe_16_Ni_2_, and (**c**) Al_82_Fe_16_Cu_2_ powders after 40, 50, and 60 h of milling, respectively; (**d**) cumulative distribution of three alloy powders.

Figure 1 shows the FE-SEM micrographs of mechanically alloyed powders, Al_82_Fe_16_Ti_2_, Al_82_Fe_16_Ni_2_, and Al_82_Fe_16_Cu_2_, after 40, 50, and 60 h of milling, respectively. In two alloys, Al_82_Fe_16_Ti_2_ and Al_82_Fe_16_Ni_2_, the particle size was about 2 to 3 µm under the SEM observation. Most particles were flattened because of the collision between powders, balls, and jar. The powder particles had a layered structure due to the repeated fracture and welding processes during the milling process. The agglomerate of small powder particles can be seen in Figure 1a,b. In the Al_82_Fe_16_Cu_2_ alloy, the particle size of powder was in the range of 2–10 µm, which implies that the welding process was more dominant. As reported in [28], Al_82_Fe_16_Ti_2_ and Al_82_Fe_16_Ni_2_ alloys are fully amorphous structures after milling for 40 and 50 h, respectively. In Al_82_Fe_16_Cu_2_ powder_,_ only partly amorphous structure was obtained even after milling for a longer time of 60 h. Particle size distribution curves of amorphous alloy samples were measured by means of laser light scattering granulometry, as shown in Figure 2. Two samples exhibited a unimodal distribution and an average particle size, d_0.5_, of 15.9 and 9.4 µm for Al_82_Fe_16_Ti_2_ and Al_82_Fe_16_Ni_2_ powders after 40 and 50 h of milling, respectively. Meanwhile, the Al_82_Fe_16_Cu_2_ alloy sample exhibited a bimodal distribution and an average particle size, d_0.5_, of 14.6 µm. Figure 2d compares the cumulative size distribution curve of three alloy powders. While the particle size distribution of Al_82_Fe_16_Ni_2_ and Al_82_Fe_16_Cu_2_ remained nearly the same in the lower size range, the cumulative size distribution curve of Al_82_Fe_16_Ti_2_ shifted rightwards, indicating that particle size shifted to a larger micron range (<30 μm at 90% volume fraction).

Table 1 listed atomic radii mismatch (in %) and enthalpies of mixing (in kJ/mole) for Al, Fe, Ni, Ti, Cu, Y, and La binary systems, according to [30,31]. The three basic empirical rules, for the achievement of high glass-forming ability, are: (1) the alloy must contain at least three components; (2) a significant atomic size difference among the main constituent elements in the alloy should be above 12%; (3) there should be a negative heat of mixing among the major constituent elements in the alloy system [32,33]. The three rules played an important role in the selection of elements for bulk metallic glasses containing rare-earth alloys fabricated by rapid quenching technique. However, there are cases where those rules do not apply, namely for metallic glass alloys containing rare-earth elements and produced by mechanical alloying, such as Al_82_Fe_16_Y_2_ and Al_82_Fe_4_Ni_4_La_10_ systems. Y and La have quite large atomic sizes, and the atomic mismatches of Al–Y and Al–La are about 21.4 and 23.9%, respectively. These alloys achieved a fully amorphous structure for a long time of milling at 100 and 350 h, respectively [12,17]. In contrast, the amorphization of Al–Fe alloys without rare-earth elements, such as Al_82_Fe_16_Ti_2_, Al_82_Fe_16_Ni_2_, and Al_82_Fe_16_Cu_2_, occurs after shorter milling periods [28]. It is evident that the atomic mismatch does not significantly influence the glass-forming ability (GFA) in Al–Fe alloys produced by the mechanical alloying technique because of the nearly similar atomic size of Ti, Ni, and Cu transition elements. However, the amorphization process varied with the mixing enthalpy values of transition elements. This could be due to the values of the different mixing enthalpy of different alloying elements in ascending orders: Al–Ti < Al–Ni < Al–Cu, as well as Fe–Ti < Fe–Ni < Fe–Cu. There is a tendency for glass formation to increase with decreasing mixing enthalpy. The Al_82_Fe_16_Ti_2_ alloy presented the most negative mixing enthalpy with all binary elements, while Ni has a small negative value of mixing enthalpy with Fe, and Cu showed a positive enthalpy of mixing with Fe. The values of mixing enthalpy may be decisive in the amorphization process.

**Figure 3:**
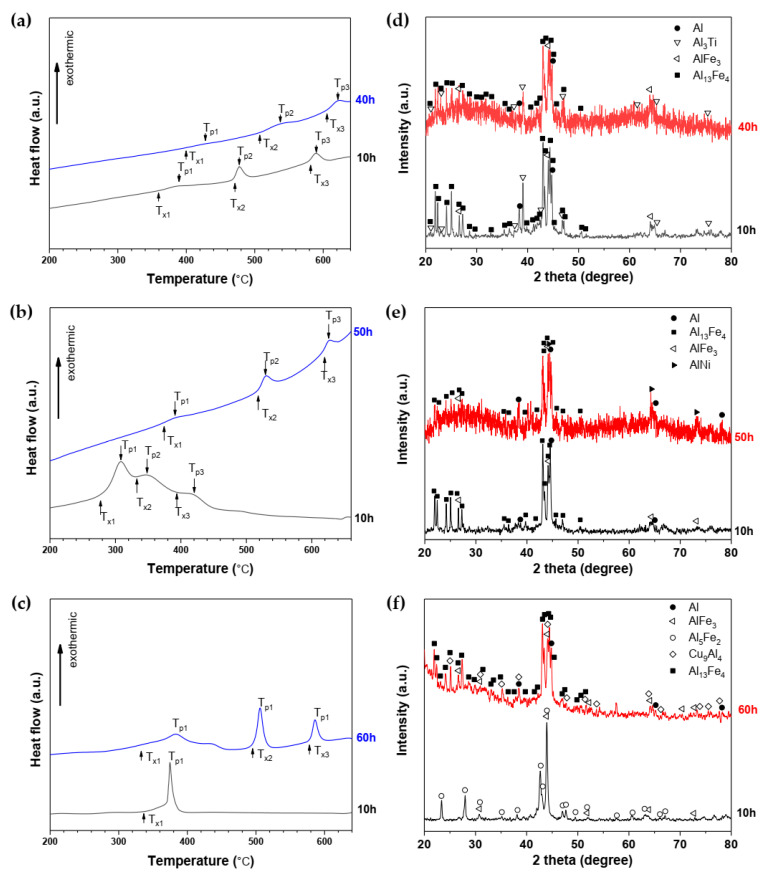
DSC curves of as-milled (**a**) Al_82_Fe_16_Ti_2_, (**b**) Al_82_Fe_16_Ni_2_, and (**c**) Al_82_Fe_16_Cu_2_ alloy powders and their XRD patterns after DSC examination of (**d**) Al_82_Fe_16_Ti_2_, (**e**) Al_82_Fe_16_Ni_2_, and (**f**) Al_82_Fe_16_Cu_2_ alloys.

In order to investigate the thermal stability of the as-milled powders, a non-isothermal DSC mode was applied. Figure 3a–c presented the DSC curves of milled powders after 10 h and amorphous states of milling, respectively. Characteristic temperatures, T_x_ and T_p_ (onset and maximum of the crystallization exothermal peak, respectively), were obtained from DSC scans of powdered samples heated at a constant heating rate of 20 K.min^−1^. Most DSC curves of three alloy powders milled at different times exhibited three exothermic peaks, except Al_82_Fe_16_Cu_2_ alloy powders milled for 10 h. The calorimetric curve recorded for this alloy presented only one peak at a temperature of about 320–420 °C. From the XRD patterns of powders milled for 10 h, there was a small broad diffuse halo of an amorphous phase together with sharpness diffraction peaks corresponding to the existence of minor volume fractions of unprocessed nanoparticles in a scattering range of 2θ between 40–50°, as presented in our previous work [28]. These nanoparticles reacted with each other to produce intermetallic phases, resulting in lower onset crystallization temperatures of three alloys. The onset temperature of amorphous Al_82_Fe_16_Ti_2_, Al_82_Fe_16_Ni_2,_ and Al_82_Fe_16_Cu_2_ alloys after milling for 40, 50, and 60 h starts at 398, 365, and 334 °C, respectively. The Al_82_Fe_16_Ti_2_ alloy had the highest onset crystallization temperature, and the Al_82_Fe_16_Cu_2_ alloy had the lowest onset temperature. This DSC profile was similar to the one reported in the literature for Al_82_Fe_18_ and Al_82_Fe_16_Y_2_ alloy [34], where the peaks are related to the exothermic effects connected with fcc-Al and intermetallic phases.

The thermal stability of three amorphous alloys can be explained by the binary mixing enthalpies of constituent elements listed in Table 1. ΔH_mix_ between Ti, Ni, Cu, and Al (solvent) is −30, −22 and −1 kJ/mole, respectively. Thus, ΔH_mix_ of Ti–Al was more negative than that of Ni–Al and Cu–Al. The crystallization onset temperature of the Al_82_Fe_16_Ti_2_ amorphous alloy was the highest value among the three alloys. The more negative the enthalpy of mixing, the larger the atomic constraint force, resulting in a higher thermal stability. The thermal stabilities of the three alloys were in the following order: Al_82_Fe_16_Ti_2_–Al_82_Fe_16_Ni_2_–Al_82_Fe_16_Cu_2_. Looking into details of the other alloys in Table 2, we can see that amorphous Al_84_Fe_16_ composition starts crystallization at 353 °C. The substitution of Fe for Al (2 at.%) in Al_84_Fe_16_, the alloy system became Al_82_Fe_18_ and the number of Al–Fe pairs increased from 16 to 18. The crystallization temperature of Al_82_Fe_18_ was raised to 375 °C. As the number of Al–Fe pairs increased, the thermal stability of Al_82_Fe_18_ alloy also increased. Similar to the above case, the substitution of Ti for Al (2 at.%) results in the crystallization onset temperature of the Al_82_Fe_16_Ti_2_ alloy, increasing to 398 °C. ΔH_mix_ of Ti–Fe (−17 kJ/mole) was more negative than that of Al–Fe (−1 kJ/mole), which means that the GFA of the Al_82_Fe_16_Ti_2_ alloy can be improved more compared to that of the Al_84_Fe_16_ alloy. However, in the Al_82_Fe_16_Ni_2_ alloy, the crystallization temperature was higher than Al_84_Fe_16_ and lower than Al_82_Fe_18_ and Al_82_Fe_16_Ti_2_ alloys. ΔH_mix_ of Ni–Fe is (−2 kJ/mole) was lower than Al–Fe (−11 kJ/mole) and Ti–Fe (−17 kJ/mole), which results in the lower crystallization temperature of the Al_82_Fe_16_Ni_2_ alloy, compared to Al_84_Fe_16_, Al_82_Fe_18_, and Al_82_Fe_16_Ti_2_ alloys. DSC curves of the Al_82_Fe_16_Cu_2_ alloy showed very broad peaks between 120 and 700 °C, similar to the Al_75_Fe_25_ alloy, as reported in [33]. The Al_82_Fe_16_Cu_2_ alloy had the lowest crystallization temperature due to its partial amorphous structure. It was found that the GFA reduced with the addition of Cu in Al_84_Fe_16_ due to the positive mixing enthalpies of Fe-Cu (+13 kJ/mole). The more negative the mixing enthalpy in the alloy, the more thermally stable an amorphous phase against solid solution and intermediate phase is [35]. Z. Zhang et al. investigated Al–Ni–RE (RE–La, Ce) alloys produced by arc melting, which exhibited a strong dependence on the size of the RE atom and negative mixing enthalpy between the constituent elements with the glass formation [36]. However, Al–Fe–2TM prepared by MA showed that the most important factor, decided by the GFA of the alloys, was a larger negative mixing enthalpy between the constituent elements.

The phase transformation of amorphous alloy powders after DSC heating was performed by using the XRD technique. The X-ray diffraction patterns of the DSC-quenched samples for as-milled Al_82_Fe_16_Ti_2_, Al_82_Fe_16_Ni_2_, and Al_82_Fe_16_Cu_2_ alloy powders are shown in Figure 3d–f. Similar phase formations were seen in Al_82_Fe_16_Ti_2_ and Al_82_Fe_16_Ni_2_ alloys after being milled for 10, 40, and 50 h. However, for Al_82_Fe_16_Cu_2_ alloy powder milled for 10 h, only Al_5_Fe_2_ and AlFe_3_ were detected from XRD patterns, while higher precipitation phases of fcc-Al, Cu_9_Al_4_, and Al_13_Fe_4_ could be only obtained from Al_82_Fe_16_Cu_2_ alloy powder milled for 60 h.

Via the thermal effect, the glassy phase was transformed into fcc-Al and intermetallic phases. The first exothermic peak around 360–390 °C represents the precipitation of crystalline α-Al on the Al matrix, which could improve the strength of the alloy and increase material performance [24]. The next two exothermal peaks mark the appearance of intermetallic phases, such as Al_13_Fe_4_, AlFe_3_, and Al_3_Ti for the Al_82_Fe_16_Ti_2_ alloy; AlNi, AlFe_3_, and Al_13_Fe_4_ for Al_82_Fe_16_Ni_2_ alloy; and Cu_9_Al_4_, Al_5_Fe_2_, AlFe_3_, and Al_13_Fe_4_ for the Al_82_Fe_16_Cu_2_ alloy. These intermetallic phases have negative formation enthalpy as the Miedema calculation [37]. The formation enthalpies of Al_13_Fe_4_, AlFe_3_, Al_3_Ti, Al_5_Fe_2_, AlNi, and Cu_9_Al_4_ are −18.052, −22.078, −39.02, −21.855, −48.424, and −13.104 kJ/mole, respectively. A mixture of Al_13_Fe_4_, Al_6_Fe, Fe_4_Y, and Al phases was reported as a crystallization product after heating the amorphous Al_82_Fe_16_Y_2_ alloy to 700 °C in the same calorimeter used in previous our work [34]. It can be realized that, for amorphous alloys with a small amount of the different transition elements or rare-earth elements obtained in the same mills, dissimilar phases crystallized during analogous heat treatment. Thus, the crystallization process of amorphous alloys with the same milling parameters can be influenced by composition. This indicates that these Al-based amorphous alloys (produced in the same mills) actually differ. The refined lattice parameters of the DSC-quenched samples were calculated using Profex software packages. It is very interesting that two Al_13_Fe_4_ and AlFe_3_ intermetallic phases were precipitated from the amorphous phase of all three alloys. Considering this, milling of these alloys resulted in the formation of solid solutions of α-Al(Fe) and α-Fe(Al), then transformed to the AlFe_3_ intermetallic phase. Additionally, in the opposite process, the crystallization of the amorphous phase, the AlFe_3_ intermetallic phase was also produced. It is noticeable that the AlFe_3_ phase had high negative formation enthalpy of −22.078 kJ/mole, so it was easy to form during crystallization. A slight difference in lattice parameters for Al_13_Fe_4_ and AlFe_3_ intermetallic phases can be seen in Table 3. A lattice expansion is observed as a result of disordering of the lattice when the iron diffusion into the aluminum lattice formed intermetallic phases during crystallization. The lattice constant of fcc-Al as a sample after DSC was similar to that in #PDF 04-0787.

## 4. Conclusions

In the present study, the thermal stability of mechanically alloying powders increased in the order of Al_82_Fe_16_Cu_2_, Al_82_Fe_16_Ni_2_, and Al_82_Fe_16_Ti_2_ alloys. The crystallization in Al_82_Fe_16_Ti_2_, Al_82_Fe_16_Ni_2_, and Al_82_Fe_16_Cu_2_ started at: 398, 365, and 334 °C, respectively. For ternary Al–Fe–TM (TM: Ti, Ni, Cu) alloys, ΔH_mix_ was the main factor deciding the amorphization process. The larger the negative mixing enthalpy between binary elements in the alloys, the faster the amorphization process was. Al_82_Fe_16_Ti_2_ amorphous alloy had the highest glass-forming ability and thermal stability among the three alloys. A considerably lower thermal stability of Al_82_Fe_16_Cu_2_ than the other alloys may arise from a system with a positive heat of mixing. A larger negative mixing enthalpy between the constituent elements was the key factor, which decided the GFA of amorphous alloys. The crystallization of these alloys occurred during the DSC process, with the transformation of the amorphous phase into fcc-Al and intermetallic phases. The diffusion of Fe in Al lattice to produce intermetallic phase Al_13_Fe_4_ and AlFe_3_ resulted in the slightly different lattice parameters compared to standard phases. The GFA and crystallization in Al–Fe–2TM metallic glasses were much more sensitive to minor alloying elements.

## Figures and Tables

**Table 1:** Atomic radii mismatch (in %) and enthalpies of mixing (in kJ/mole) for Al, Fe, Ni, Ti, Cu, Y, and La binary systems [30].

Element	Al	Fe	Ti	Ni	Cu	Y	La
**Al**	-	13 (%)	2.7 (%)	12.5 (%)	10 (%)	21.4 (%)	23.9 (%)
**Fe**	−11 (kJ/mole)	-	15.6 (%)	0.8 (%)	3.1 (%)	31.8 (%)	34 (%)
**Ti**	−30 (kJ/mole)	−17 (kJ/mole)	-	14.9 (%)	12.9 (%)	19.2 (%)	21.8 (%)
**Ni**	−22 (kJ/mole)	−2 (kJ/mole)	−35 (kJ/mole)	-	2.3 (%)	31.3 (%)	33 (%)
**Cu**	−1 (kJ/mole)	+4 (kJ/mole)	−9 (kJ/mole)	+4 (kJ/mole)	-	29.6 (%)	31.9 (%)
**Y**	−38 (kJ/mole)	−1 (kJ/mole)	+15 (kJ/mole)	−31 (kJ/mole)	−22 (kJ/mole)	-	3 (%)
**La**	−38 (kJ/mole)	+5 (kJ/mole)	+20 (kJ/mole)	−27 (kJ/mole)	−21 (kJ/mole)	+20 (kJ/mole)	-

**Table 2:** Crystallization temperatures and phase compositions of Al-Fe alloys produced by a planetary ball mill.

Alloys	Phase after MA	Crystallization Temperatures, (°C)							Crystallization Phases	Ref
		T_x1_	T_p1_	T_x2_	T_p2_	T_x3_	T_p3_	T_x4_		
Al_84_Fe_16_	amorphous	353	-	450	-	511	-	590	Al, Al_13_Fe_4_, Al_6_Fe	[34]
Al_82_Fe_18_	amorphous	380	-	491	-	579	-	-	Al, Al_13_Fe_4_	[34]
Al_82_Fe_16_Y_2_	amorphous	382	-	486	-	584	-	-	Al, Al_6_Fe, Fe_4_Y, Al_13_Fe_4_	[34]
Al_82_Fe_16_Ti_2_	amorphous	398	424	507	535	605	623	-	Al, Al_3_Ti, AlFe_3_, Al_13_Fe_4_	This work
Al_82_Fe_16_Ni_2_	amorphous	365	393	516	530	617	627	-	Al, Al_13_Fe_4_, AlFe_3_, AlNi	This work
Al_82_Fe_16_Cu_2_	Partly amorphous	334	382	495	506	576	586	-	Al, Al_13_Fe_4_, AlFe_3_, Al_5_Fe_2_, Cu_9_Al_4_	This work

**Table 3:** Refined lattice parameters of crystallization phases formed from DSC-quenched Al_82_Fe_16_Ti_2_, Al_82_Fe_16_Ni_2_, and Al_82_Fe_16_Cu_2_ alloy powders.

Sample	Phase	ICDD/JCPDS ID *	Lattice Parameters (nm)	CIF ID **	Refined Lattice Parameters (nm)		Formation Enthalpy, kJ/mol
**Al_82_Fe_16_Ti_2_**					MA 10 h	MA 40 h	
Cubic, *Fm−3m* (*225*)	Al	04-0787	*a* = 0.40494		*a* = 0.40584	*a* = 0.4049	
Monoclinic *C2/m* (*12*)	Al_13_Fe_4_	29-0042	*a* = 1.5489 *b* = 0.8083 *c* = 1.2476 β = 107.7	ICSD_151129	*a* = 1.5498 *b* = 0.8089 *c* = 1.2501 β = 107.93	*a* = 1.5511 *b* = 0.8092 *c* = 1.2527 β = 108.15	−18.052
Cubic *Fm-3m* (*225*)	AlFe_3_	45-1203	*a* = 0.57934	mp-2018	*a* = 0.5765	*a* = 0.5803	−22.078
Tetragonal *I4/mmm* (*139*)	Al_3_Ti	37-1449	*a* = 0.38537 *c* = 0.85839	mp-542915	*a* = 0.3851 *c* = 0.8602	*a* = 0.3852 *c* = 0.8609	−39.020
**Al_82_Fe_16_Ni_2_**					MA 10 h	MA 50 h	-
Cubic *Fm-3m* (*225*)	Al	-	*a* = 0.40494	-	*a* = 0.4053	*a* = 0.4049	-
Monoclinic *C2/m* (*12*)	Al_13_Fe_4_	29-0042	*a* = 1.5489 *b* = 0.8083 *c* = 1.2476 β = 107.7	ICSD_151129	*a* = 1.5462 *b* = 0.8118 *c* = 1.2489 β = 107.81	*a* = 1.5495 *b* = 0.8084 *c* = 1.2491 β = 107.89	-
Cubic *Fm-3m* (*225*)	AlFe_3_	50-0955	*a* = 0.58152	mp-2018	*a* = 0.5747	*a* = 0.5803	-
**Al_82_Fe_16_Cu_2_**					MA 10 h	MA 60 h	-
Cubic *Fm-3m* (*225*)	Al	-	*a* = 0.40494	-	-	*a* = 0.4050	-
Monoclinic *C2/m* (*12*)	Al_13_Fe_4_	29-0042	*a* = 1.5489 *b* = 0.8083 *c* = 1.2476 β = 107.7	ICSD_151129	-	*a* = 1.5515 *b* = 0.8094 *c* = 1.2521 β = 107.86	-
Cubic *Fm-3m* (*225*)	AlFe_3_	50-0955	*a* = 0.58152	mp-2018	*a* = 0.5803	*a* = 0.5771	-
Orthorhombic *Cmcm* (*63*)	Al_5_Fe_2_	47-1435	*a* = 0.76486 *b* = 0.64131 *c* = 0.42165	COD_2101159	*a* = 0.7620 *b* = 0.6424 *c* = 0.4204	-	−21.855
Cubic *P-43m* (*215*)	Cu_9_Al_4_	24-0003	*a* = 0.87027	mp-593	-	*a* = 0.8789	−13.104

* id of materials taken from ICDD/JCPDS were indexed in MDI Jade 6.5. ** id of materials were taken from materialsproject.org, www.crystallography.net (accessed on 7 August 2020), and icsd.products.fiz-karlsruhe.de, which were then converted to structure files used in Profex software.

## Data Availability

Data is contained within the article.
